# Unravelling copper effect on the production of varietal thiols during Colombard and Gros Manseng grape juices fermentation by *Saccharomyces cerevisiae*

**DOI:** 10.3389/fmicb.2023.1101110

**Published:** 2023-04-25

**Authors:** Gabriel Dournes, Thierry Dufourcq, Lucas Suc, Aurélie Roland, Jean-Roch Mouret

**Affiliations:** ^1^UMR SPO, Univ Montpellier INRAE, Institut Agro, Montpellier, France; ^2^Institut Français de la Vigne et du Vin, Pôle Sud-Ouest, Caussens, France

**Keywords:** copper, thiol precursors, varietal thiol, fermentation, Total thiol, Colombard, Gros Manseng

## Abstract

Nowadays the rapidly increasing organic vineyard management with the utilization of copper as sole fungal control pesticide against downy mildew raises once again the question of copper impact on varietal thiols in wine. For this purpose, Colombard and Gros Manseng grape juices were fermented under different copper levels (from 0.2 to 3.88  mg/l) to mimic the consequences in must of organic practices. The consumption of thiol precursors and the release of varietal thiols (both free and oxidized forms of 3-sulfanylhexanol and 3-sulfanylhexyl acetate) were monitored by LC–MS/MS. It was found that the highest copper level (3.6 and 3.88  mg/l for Colombard and Gros Manseng respectively) significantly increased yeast consumption of precursors (by 9.0 and 7.6% for Colombard and Gros Manseng respectively). For both grape varieties, free thiol content in wine significantly decreased (by 84 and 47% for Colombard and Gros Manseng respectively) with the increase of copper in the starting must as already described in the literature. However, the total thiol content produced throughout fermentation was constant regardless of copper conditions for the Colombard must, meaning that the effect of copper was only oxidative for this variety. Meanwhile, in Gros Manseng fermentation, the total thiol content increased along with copper content, resulting in an increase up to 90%; this suggests that copper may modify the regulation of the production pathways of varietal thiols, also underlining the key role of oxidation. These results complement our knowledge on copper effect during thiol-oriented fermentation and the importance of considering the total thiol production (reduced+oxidized) to better understand the effect of studied parameters and differenciate chemical from biological effects.

## 1. Introduction

Varietal thiols are interesting aromas for white wines, especially as they are imparting notes of grapefruit with 3-sulfanylhexanol (3SH) (Tominaga et al., 1998a), passion fruit with 3-sulfanylhexyl acetate (3SHA) (Tominaga et al., 1996) as well as blackcurrant bud with 4-methyl-4-sulfanyl-pentanone (4MSP) (Darriet et al., 1995). These aromas are produced during fermentation by yeast from odorless precursors found in grapes of different varieties such as Sauvignon blanc, Colombard and Gros Manseng (Roland et al., 2011a; Dournes et al., 2022).

The precursors of 3SH, which is the most ubiquitous varietal thiol, are either bound to glutathione (Peyrot Des Gachons et al., 2002; Roland et al., 2010a; Thibon et al., 2016), dipeptides (Capone et al., 2011; Bonnaffoux et al., 2017) or cysteine (Tominaga et al., 1998b). These precursors once consumed by yeast will enter a degradation pathway that looks like in every way the xenobiotic detoxification pathway, which will convert them to Cys3SH (Bonnaffoux et al., 2018); this precursor will then be cleaved into 3SH and pyruvate by the action of an enzyme with β-lyase activity such as Irc7p (Thibon et al., 2008) or Str3p (Holt et al., 2011). This polyfunctional thiol (thiol and alcohol functions) can then enter the same route as the higher alcohols produced by yeast and be acetylated on the alcohol function, mainly by Atf1p, to produce 3SHA (Swiegers et al., 2006).

Varietal thiol formation during fermentation is a key process for the production of wines with powerful aromatic profiles. Over the years, many parameters have been identified to regulate thiol final concentration in wine. Among them, yeast strain could be the most important, as Irc7p exists under two different forms of which only one is active (Roncoroni et al., 2011). Secondly, thiol precursors consumption and degradation are controlled by yeast nitrogen catabolite repression (NCR) (Subileau et al., 2008; Thibon et al., 2008) and therefore by the nitrogen status of the must. Thirdly, as varietal thiols are readily reactive and particularly prone to oxidation, oxygen management is particularly important at the end of fermentation (Blanchard et al., 2004; Lopes et al., 2009; Roland et al., 2010c).

Copper has been used on grapevine for more than 120 years for its antifungal properties (Prillieux, 1887). Its utilization was the main line of defense against fungal parasites such as downy mildew (*Plasmopara viticola*), it was remplaced in the early 50s with synthetic alternatives. However, for grapevine organic cultivation, theses synthetic fungicides are forbidden, which implies the exclusive use of copper as sole fungicide. In organically managed vineyards, copper concentration in must can reach up to 15 mg/l (García-Esparza et al., 2006). According to the Compendium of International Methods of Analysis of the Organisation Internationale de la Vigne et du Vin (OIV), the maximum acceptable limit for copper in wine is 1 mg/l (Organisation Internationale de la Vigne et du Vin (OIV), 2009). In the literature, some reviews indicate that ‘normal wines’ contain 0.7 to 0.8 mg/l of copper (Claus, 2020), while recommended values range from 0.3 mg/l to 0.5 mg/l in wine (Clark and Scollary, 2000). For organic wines, one study described levels of copper in different wines such as Chardonnay (462.3 ± 2.9 μg/l), Uva di Troia (267.0 ± 4.0 μg/l), Primitivo (255.0 ± 8.6 μg/l) and Negroamaro (116.7 ± 0.6 μg/l) (Provenzano et al., 2010). Surprisingly, the levels of copper in organic wines seem to be very moderate and in line with OIV regulations.Copper impact has been studied soon in the early days of varietal thiols science (Darriet et al., 2001). It was shown that copper spraying on parcels of Sauvignon blanc, Cabernet Sauvignon and Merlot significantly decreased 3SH content in wine, between 89 and 37% (for 1 copper treatment) and between 94 and 63% (for two copper treatments). The same work also demonstrated limited to no impact of foliar copper spraying on wine varietal thiol content, traducing no assimilation of copper by the plant through aerial organs. At that time, knowledge about varietal thiols, their precursors and their fates was limited and conclusions were only incomplete. Since then, it was shown that copper spraying at the vineyard could affect the precursors content of specific varieties (Dournes et al., 2022) with 30% less precursors in copper -reated Gros Manseng grapes compared to their control while Colombard remained unaffected, revealing a possible biological effect of copper in the vineyard.

**Figure 1:**
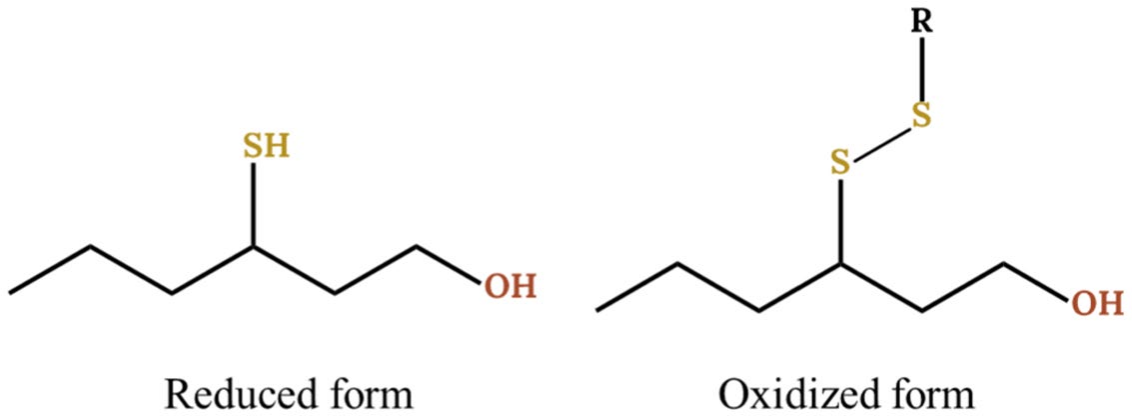
Reduced and oxidized form of 3SH with R being an undefined carbon chain.

Due to thiol reactivity, their fates are quite various, including the reaction with either acetaldehyde to form oxathiane (Chen et al., 2018; Wang et al., 2020), polyphenols to form thioether adducts (Nikolantonaki et al., 2012) or other thiols to form di- and poly- sulfides (Roland et al., 2016; Dekker et al., 2020). Until 2016 and the development of the N-phenylmaleimide (NPM) method by Roland et al. (2016), only the reduced form of thiols was studied, providing only an incomplete view of these aromas (Figure 1). Furthermore, the same work showed the involvement of copper sulfate in the oxidation of thiols into disulfide with up to 90% of 3SH being transformed. It is therefore necessary to analyse the thiols forms in their entirety in order to fully understand the impact of copper during winemaking.

In this work, the impact of copper on the consumption of thiol precursors by *Saccharomyces cerevisiae* was studied during the fermentation of Colombard and Gros Manseng grape juices as well as its impact on the content of free and oxidized 3SH produced in the corresponding wines.

## 2. Materials and methods

### 2.1. Chemicals

UPLC/MS grade water, methanol and formic acid (>99.98%) were purchased from Biosolve Chimie, Dieuze, France; deuterium (99.8% D), dithiotreitol (98%), (*E*)-2-hexenal (98%), 2-hexyn-1-ol (≥97%), Boc-Cys-OH (≥98.5%), (Boc-Cys-OH)_2_ (≥98%), ethanol-OD (≥99%), hydrochloric acid (37%), L-glutathione reduced (98%), Lindlar catalyst, manganese(IV) oxide (≥99%), mesityl oxide (≥90%), mesityl oxide d_10_ (>98% D), triethylamine (≥99%) were purchased from Sigma-Aldrich, St Quentin Fallavier, France, and H-Cys-Gly-OH (98%), H-Glu(Cys-OH)-OH (98%)from Bachem, Bubendorf, Suisse; triethylsilane (≥98%) was obtained from TCI Europe, Zwijndrecht, Belgium; while di-sodium hydrogen phosphate (≥99%), sodium carbonate (≥99%), sodium dihydrogen phosphate dihydrate (≥99%)and trifluoroacetic acid (≥99.9%) were purchased from Carl Roth, Karlsruhe, Germany.

### 2.2. Grape sampling

Two plots grown with either Colombard or Gros Manseng were selected in the region of Côtes de Gascogne in France during the 2020 vintage. At harvest, 100 kg of grapes were handpicked for vinifications.

### 2.3. Mini Vinifications

The 100 kg of grapes were received and processed immediately upon reception at the wine cellar. The first step consisted in destemming and crushing with Bucher DeltaE1 (Bucher Vaslin, Chalonnes sur Loire, France). The resulting crushed berries were sulfited at 30 mg/l using a 10% bisulfite liquid solution (Solution 10, Laffort, Bordeaux, France) and left to macerate for 15 h at 4°C. After maceration, crushed grapes were pressed under inert gas using a modified pneumatic press (Marchisio, Vertova, Italy) for 600 s at 2 bars with another dose of 40 mg/l of sulfites during this process. Grape juice (80 l) was then split equally into two 50 l plastic barrels per variety and cooled at 4°C for 72 h to clarify to 150 nephelometric turbidity units (NTU) measured with a 2,100 AN IS turbidimeter (Hach, Loveland, USA). For each variety, the clarified musts from the two barrels were pooled and homogenized before being transferred into LeParfait® 3 l jar previously inerted with dry ice.

The jars were filled with 2.5 l of must corresponding to each grape variety juices and stored in a controlled room at 18°C. The three copper levels (+0.41, +0.8 and + 3.45 mg/l of Cu) were achieved with the addition of 0.5, 1, 4.25 ml of Sulfiredox ® solution (Laffort, Bordeaux, France) respectively. These four conditions were denominated ‘Control’, ‘Cu1’, ‘Cu2’, ‘Cu3’.

Fermentation started with the inoculation of 200 mg/l of rehydrated active dried *Saccharomyces cerivisiae* yeast (Zymaflore X5, Laffort, Bordeaux, France) and the addition of 200 mg/l of Vivactiv Arôme (Sofralab, Magenta, France) and 200 mg/l of Go-Ferm® protect (Lallemand, Canada). Fermentations further received 100 mg/l of Vitaferment® (Lamothe-Abiet, Canéjan, France) at inoculation time and three days after. All fermentations were carried out in triplicate at 18°C; density was monitored every day and fermentations were stopped at density < 0.92.

### 2.4. Copper analysis

Copper concentration in musts was analyzed with EnzytecColor COPPER (R-Biopharm, Darnstadt, Germany) on a Gallery Discrete Analyzer (ThermoFisher, Waltham, MA, USA) (Dournes et al., 2022).

### 2.5. Oenological parameters

Glucose, fructose, ethanol, glycerol, acetate and succinate contents were determined using a 1,260 Infinity HPLC (Agilent™ Technologies, Santa Clara, California) with a separation performed using a Phenomenex Rezex ROA-Organic Acid H+ (8%) column (8 μm, 300 mm x 7.8 mm) at 60°C. Detection was performed using a refractive index and UV detectors. The mobile phase was composed of H_2_SO_4_ (2.5 mM) in water and the separation was made with a constant flow rate of 0.6 ml/min (Rollero et al., 2015).

A Gallery Discrete Analyzer (ThermoFisher, Waltham, MA, USA) was used to analyze amino acids and ammonium using a Primary Amino Nitrogen Assay Kit and Ammonia Assay Kit, respectively, (Megazyme, Wicklow, Ireland).

### 2.6. Chemical syntheses of natural and deuterated thiol precursors

3-*S*-glutathionylhexan-1-ol (G3SH/G3SH-d_2_) (Roland et al., 2010a), 4-*S*-glutathionyl-4-methylpentan-2-one (G4MSP/ G4MSP-d_10_) (Roland et al., 2010b), 3-*S*-cysteinylhexan-1-ol (Cys3SH/Cys3SH-d_2_), 4-*S*-cysteinyl-4-methylpentan-2-one (Cys4MSP/Cys4MSP-d_6_) (Dagan, 2006), 3-*S*-(cysteinylglycine)-hexan-1-ol (CysGly3SH) (Fedrizzi et al., 2012), 3-*S*-(glutamylcysteinyl)-hexan-1-ol (GluCys3SH) (Fedrizzi et al., 2012) as well as 3SH-d_3_ and 3SHA-d_3_ (Kotseridis et al., 2000) were synthesized according to published methods. 3-*S*-(cysteinylglycine)-hexan-1-ol-d_2_ (CysGly3SH-d_2_) and 3-*S*-(glutamylcysteinyl)-hexan-1-ol-d_2_ (GluCys3SH-d_2_) were synthesized using 2-hexenal-d_2,_ which was obtained by 2-hexynol deuteration as reported by Roland et al. (2016). NMR characterizations of CysGly3SH-d_2_ and GluCys3SH-d_2_ were consistent with those reported by Bonnaffoux et al. (2017) (data not shown).

Synthetic and natural compounds were characterized and quantified with ^1^H NMR (data not shown).

### 2.7. Analysis of thiol precursors By stable isotope dilution assay (SIDA) and UPLC-MS/MS

Aforementioned precursors of 3SH and 4MSP were analyzed by SIDA-UPLC-MS/MS through direct injection of Colombard and Gros Manseng grape must as previously reported (Bonnaffoux et al., 2017) modified as follows: the analytical system consisted of a 1,290 Infinity II UHPLC (Agilent Technologies, Santa Clara, CA, USA) hyphenated to a 6470B Agilent Triple Quadrupole. Analytes were separated on a Hypersil gold column (1.9 μm, 100 mm x 2.1 mm) (ThermoFisher, Waltham, MA, USA) with a 40°C oven temperature and a 15 min total run time. The mobile phases were composed of (A) water or (B) methanol each with 0.1% formic acid. The gradient started at 5% of B for 1 min, was increased to 35% over 9 min, to 98% in 3 min, held for 2 min, then to 5% in 10 s and held for 2 min at a flow rate of 0.6 ml/min. Source parameters were as follows: gas temp was 230°C, gas flow was 4 l/min, nebulizer at 3.79 bar, sheath gas temp at 400°C, sheath gas flow at 11 l/min, capillary voltage at 3000 V in positive mode, nozzle voltage at 0 V and positive Delta EMV set to 400 V. Ionization was carried out using positive electrospray (ESI+) and detection was made using Multiple Reaction Monitoring (MRM). Quantification and qualification transitions were described in Table S2.

### 2.8. Analysis of thiols By stable isotope dilution assay (SIDA) and UPLC-MS/MS

Reduced and total 3SH were analyzed by SIDA-UPLC-MS/MS following a previously established method (Roland et al., 2016) based upon derivatisation with N-phenylmaleimide and tris(2-carboxyethyl)phosphine (TCEP) reduction for total thiols.

The analytical system consisted of a 1,290 Infinity II UHPLC hyphenated to a 6470B Triple Quadrupole (Agilent Technologies, Santa Clara, CA, USA). Analytes were separated on a Hypersil gold column (1.9 μm, 100 mm x 2.1 mm) (ThermoFisher, Waltham, MA, USA) with a 40°C oven temperature and total run time of 10 min. The mobile phases were composed of (A) water with 0.1% formic acid and (B) methanol with 0.1% formic acid. The gradient started at 20% of B, was increased to 80% over 4 min, to 99% in 2 min, held for 1 min, then decreased to 20% in 1 min and held for 2 min at a flow rate of 0.5 ml/min. Source parameters were as follows: gas temp was 230°C, gas flow was 10 l/min, nebulizer at 3.79 bar, sheath gas temp at 300°C, sheath gas flow at 12 l/min, capillary voltage at 3000 V in positive mode, nozzle voltage at 1000 V and positive Delta EMV set to 200 V. Ionization was carried out using positive electrospray (ESI+) and detection was made using Multiple Reaction Monitoring (MRM). Quantification and qualification transitions are described in Table S2.

### 2.9. Statistical analysis

Statistical analyses (*t*-test) were performed with RStudio software (version 3.6.2). Copper effect on fermentation metabolites for each variety was analyzed using an analysis of variance (ANOVA).

## 3. Results and discussion

### 3.1. Composition of The grape musts used for fermentation

The musts used in this study originated from the two Colombard and Gros Manseng varieties cultivated in Côtes de Gascogne region without copper. This conventional cultivation practice was selected to produce a must unaffected by organic practices, offering the possibility to design the copper range as desired. Sugar, assimilable nitrogen and copper content values (Table 1) were found to be within the range of the natural diversity encountered within these varieties for conventional cultivation practices (Dournes et al., 2022).

**Table 1:** Basic oenological parameters and 3SH precursors of Colombard and Gros Manseng musts.

**Variety**	**Colombard**	**Gros Manseng**
**Fructose (g/L)**	103 ± 10	121 ± 12
**Glucose (g/L)**	105 ± 10	112 ± 11
**Ammonium (mgN/L)**	69 ± 7	109 ± 11
**Amino acids (mgN/L)**	114 ± 11	164 ± 16
**Copper (mg/L)**	0.20 ± 0.02	0,40 ± 0.04
**G3SH (μg/L)**	109 ± 1	336 ± 3
**γ-GluCys3SH (μg/L)**	n.d.	n.d.
**CysGly3SH (μg/L)**	13.1 ± 0.1	13.6 ± 0.1
**Cys3SH (μg/L)**	5.9 ± 0.3	11.2 ± 0.6

Measure error was standardized at 10% for all analyses except for the precursors for which error represents the relative standard error of the method.

On the precursors side, it can be noted that G3SH at 109 μg/l for Colombard is lower than the previously established composition for this variety at 129–446 μg/l (Dournes et al., 2022). As to Gros Manseng, the 336 μg/l G3SH average is on the higher side of the range (145–454 μg/l) for this variety, also based on Dournes et al. work. These authors established that G3SH represented the majority of the precursor forms (more than 90%), which was also the case here especially for Gros Manseng grapes. Interestingly, it can be noted the presence of CysGly3SH at 13.1 and 13.6 μg/l in both grape varieties. These quantities are in the same order of magnitude as those found in Sauvignon blanc that vary from 0 to 20 μg/l (Capone et al., 2011) or from 5,8 to 28,3 μg/l in macerated Sauvignon blanc (155 samples) with an 11,9 μg/l average. Precursors of 4MSP and GluCys3SH were not detected in the musts. Both musts presented natural concentration of copper at 0.2 and 0.4 mg/l for Colombard and Gros Manseng, respectively.

### 3.2. Monitoring of fermentations under copper stress

Copper is a necessary microelement for all organisms, however, at high concentration this metal becomes toxic to cells (Avery et al., 1996). In winemaking, a high copper concentration has been shown to be responsible for longer fermentations and a lower alcohol production (Ferreira et al., 2006). This work also showed a strain-dependent effect as the copper concentration used (15.89 mg/l) resulted in a particularly important impairment of yeast growth for VIN13, a varietal thiol-optimized yeast strain.

While high doses of copper are used to study the impact of copper on primary metabolites and microbiological aspects during fermentation, no study has, to our knowledge, covered the impact of oenological copper doses on secondary metabolites such as varietal thiols and their precursors during fermentation. To investigate this, both Colombard and Gros Manseng juices were spiked with a CuSO_4_·5H_2_O (25 g/l) solution to obtain 3 different copper concentrations plus a control experiment without any addition. For both varieties, the different levels of spiking were as follows:

- The first condition consisted in the addition of 0.5 ml of the copper sulfate solution for the 2.5 l of must to obtain a final concentration of 0.6 mg/l of copper, corresponding to a tipping point in term of thiols in these matrices (Dufourcq et al., 2010).
- The second condition was a final copper concentration of 1 mg/l.
- Finally, adding 4,25 of the copper solution resulted in a 3.6 mg/l concentration, which was the highest measured copper content in musts in Côtes de Gascogne during this vintage.

**Figure 2:**
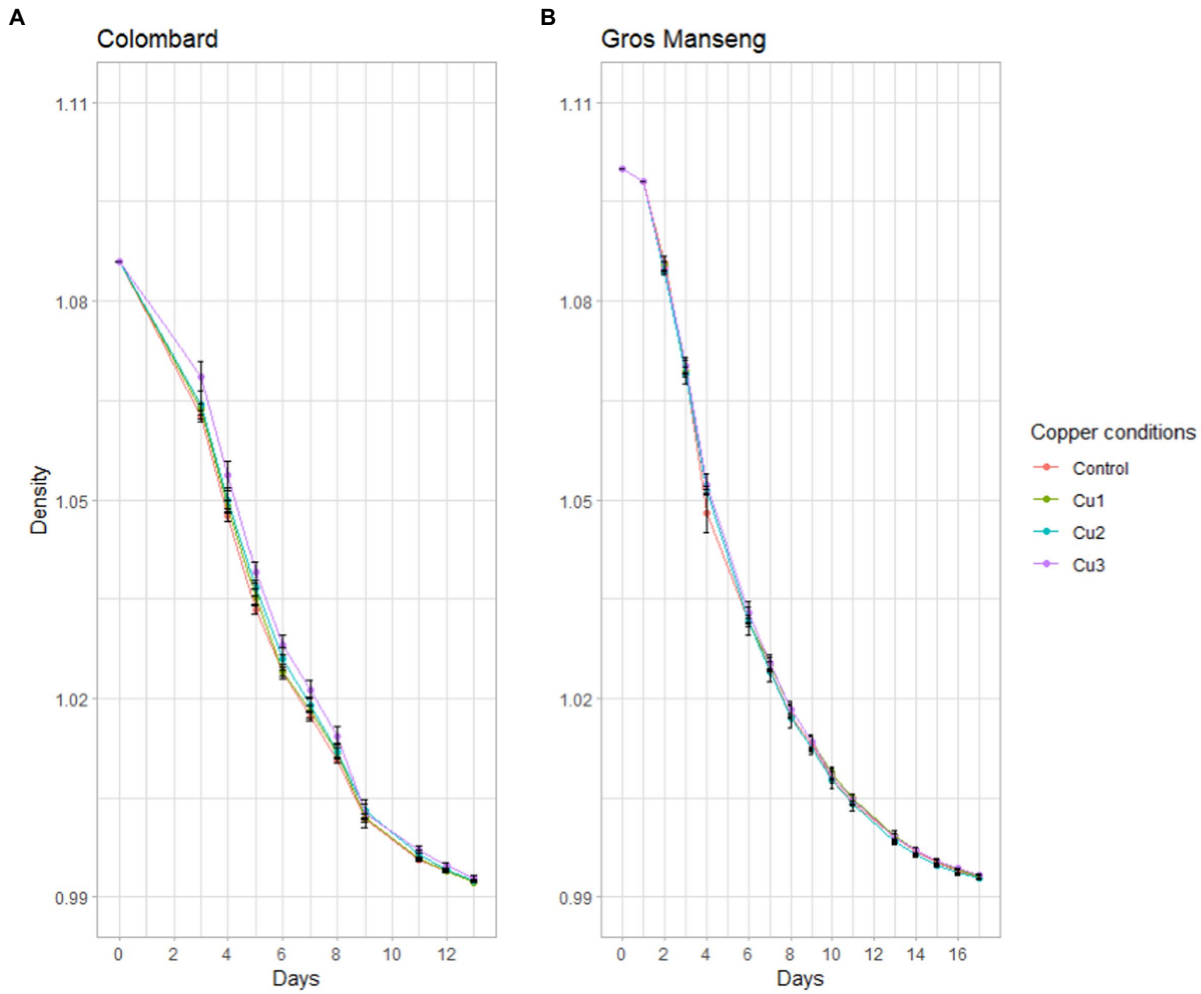
Fermentation kinetics expressed as density decrease. **(A)** Colombard and **(B)** Gros Manseng fermentation. Each point represents the mean of a biological triplicate and errors bars represent the standard error. Copper conditions in mg/L: Colombard control (0.2), Cu1 (0.6), Cu2 (1), Cu3 (3.6); Gros Manseng control (0.4), Cu1 (0.8), Cu2 (1.2), Cu3 (3.9).

Gros Manseng conditions were based on the same additions. Fermentations advancement was monitored with density measurements (Figure 2).

All conditions led to the same density decrease throughout fermentation with no difference at any point between the conditions. For both varieties, the different copper conditions led to fermentations with identical overall behavior. These results are in accordance with published results (Gava et al., 2016) where the fermentation parameters of strain X5 were unaffected by copper concentration and formulation with the exception of a slightly longer lag phase for copper oxychloride (23.40 h) compared to copper hydroxide (22.44 h), while copper sulfate was intermediate (23.03 h). For other strains such as Anaferm Riesling, fermentation parameters remained unchanged up to 25 mg/l of copper while Oenoferm X-trem showed slower fermentation from 10 mg/l upwards. Other works have reported that fermentation kinetics were differently affected at 15 mg/l of copper (Ferreira et al., 2006), suggesting that yeast strain was the most important factor in copper resistance, as reported later (Capece et al., 2016, 2017).

In this work, neither fermentation kinetics nor the main fermentation metabolites (Table 2) were affected by the copper concentrations used. For each copper modality of the two grape varieties, none of the final concentrations of the central carbon metabolism were different compared to the control. It can be assumed that there was no effect of copper at these concentrations on the analyzed elements of the yeast central metabolism. However, other metabolites, such as acetaldehyde, that are known to be influenced by copper (Capece et al., 2017; Zimdars et al., 2019) were not covered in this study.

**Figure 3:**
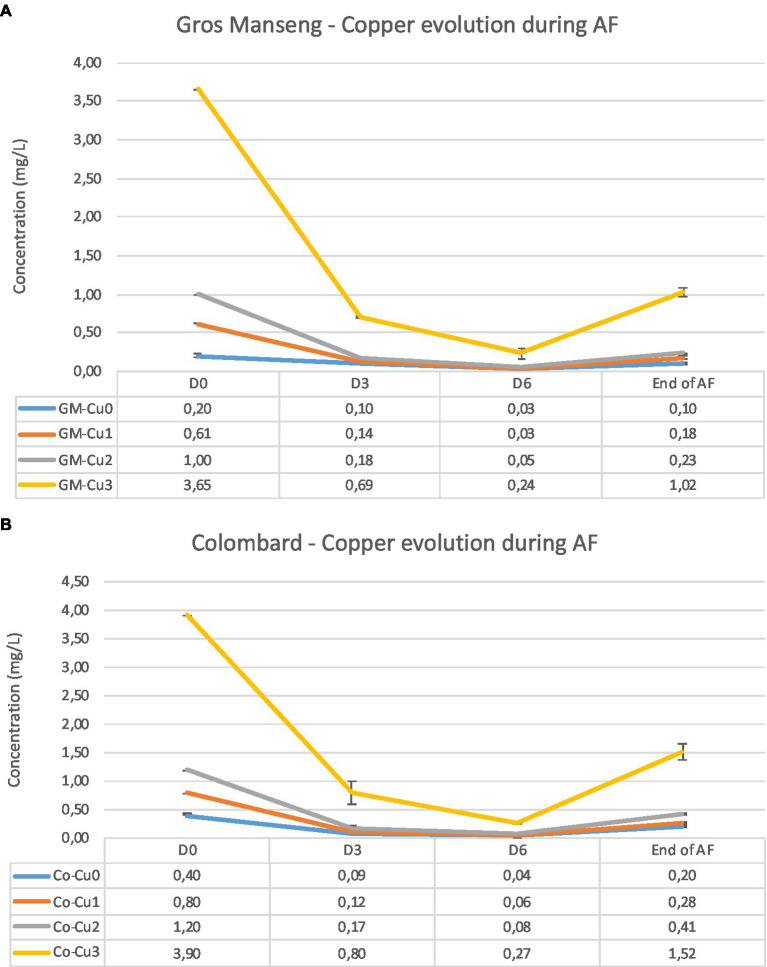
Copper consumption in Gros Manseng **(A)** and Colombard **(B)** grape juice during alcoholic fermentation. Monitoring has been made at 4 specific sampling dates: D0: before yeast inoculation; D3: 3 days after yeast inoculation; D6: 6 days after yeast inoculation and End of AF.

Finally, we monitored the copper consumption during the alcoholic fermentation and we observed that for the experiments Cu0, Cu1 and Cu2, the residual copper content ranged from 0.10 to 0.41 mg/L, whatever the type of grape juice (Table 2). For the Cu3 experiments, the residual concentrations were higher, reaching up to 1.52 mg/L. Under our conditions, the copper consumption ranged from 52 to 77% and it seems that there is no difference between the Cu1, Cu2 and Cu3 experiments, suggesting that the copper assimilation could not be dependent on the initial level of copper. In terms of kinetics, copper consumption was faster during the first 3 days, with minimal levels reached 6 days after yeast inoculation with 92% of copper consumed on average for all experiments (Figure 3). After 6 days, copper was released into the medium, probably due to cell lysis at the end of the AF.

**Table 2:** Concentration of the main fermentation metabolites at the end of fermentation.

**Variety**	**Modality**	**Ethanol (g/L)**	**Fructose (g/L)**	**Glucose (g/L)**	**Glycerol (g/L)**	**Acetate (g/L)**	**Succinate (g/L)**	**Copper (mg/L)**	**Average copper consumption (%)** *
**Colombard**	**Control**	95.8 ± 0.4	3.1 ± 0.1	n.d.	5.1 ± 0.5	0.26 ± 0.04	2.5 ± 0.3	0.20 ± 0.03	51
	**Cu1**	95.9 ± 1.2	2.5 ± 0.1	n.d.	5.8 ± 0.1	0.28 ± 0.01	2.7 ± 0.1	0.28 ± 0.01	65
	**Cu2**	95.3 ± 1.2	2.5 ± 0.2	n.d.	5.7 ± 0.2	0.27 ± 0.01	2.7 ± 0.1	0.41 ± 0.02	65
	**Cu3**	91.6 ± 3.3	2.7 ± 0.3	n.d.	5.5 ± 0.3	0.27 ± 0.02	2.6 ± 0.1	1.52 ± 0.13	61
**Gros Manseng**	**Control**	111.0 ± 0.7	6.2 ± 0.4	0.5 ± 0.1	8.3 ± 0.1	0.76 ± 0.01	7.5 ± 0.2	0.10 ± 0.02	52
	**Cu1**	112.9 ± 0.4	5.6 ± 0.1	0.4 ± 0.2	8.2 ± 0.1	0.75 ± 0.02	7.5 ± 0.1	0.18 ± 0.02	70
	**Cu2**	112.5 ± 0.3	5.9 ± 0.4	0.4 ± 0.1	8.2 ± 0.1	0.75 ± 0.01	7.5 ± 0.1	0.23 ± 0.01	77
	**Cu3**	112.6 ± 0.4	5.6 ± 0.2	0.4 ± 0.1	8.3 ± 0.1	0.75 ± 0.01	7.5 ± 0.1	1.02 ± 0.05	72

For each value, are presented the average and the standard deviation for the fermentation triplicate. A statistical test using ANOVA produced no significant difference between modalities in each variety. Copper conditions in mg/L: Colombard control (0.2), Cu1 (0.6), Cu2 (1), Cu3 (3.6); Gros Manseng control (0.4), Cu1 (0.8), Cu2 (1.2), Cu3 (3.9).

* data referred to the consumption of copper at the end of AF.

### 3.3. Precursors consumption By The yeast during alcoholic fermentation

**Figure 4:**
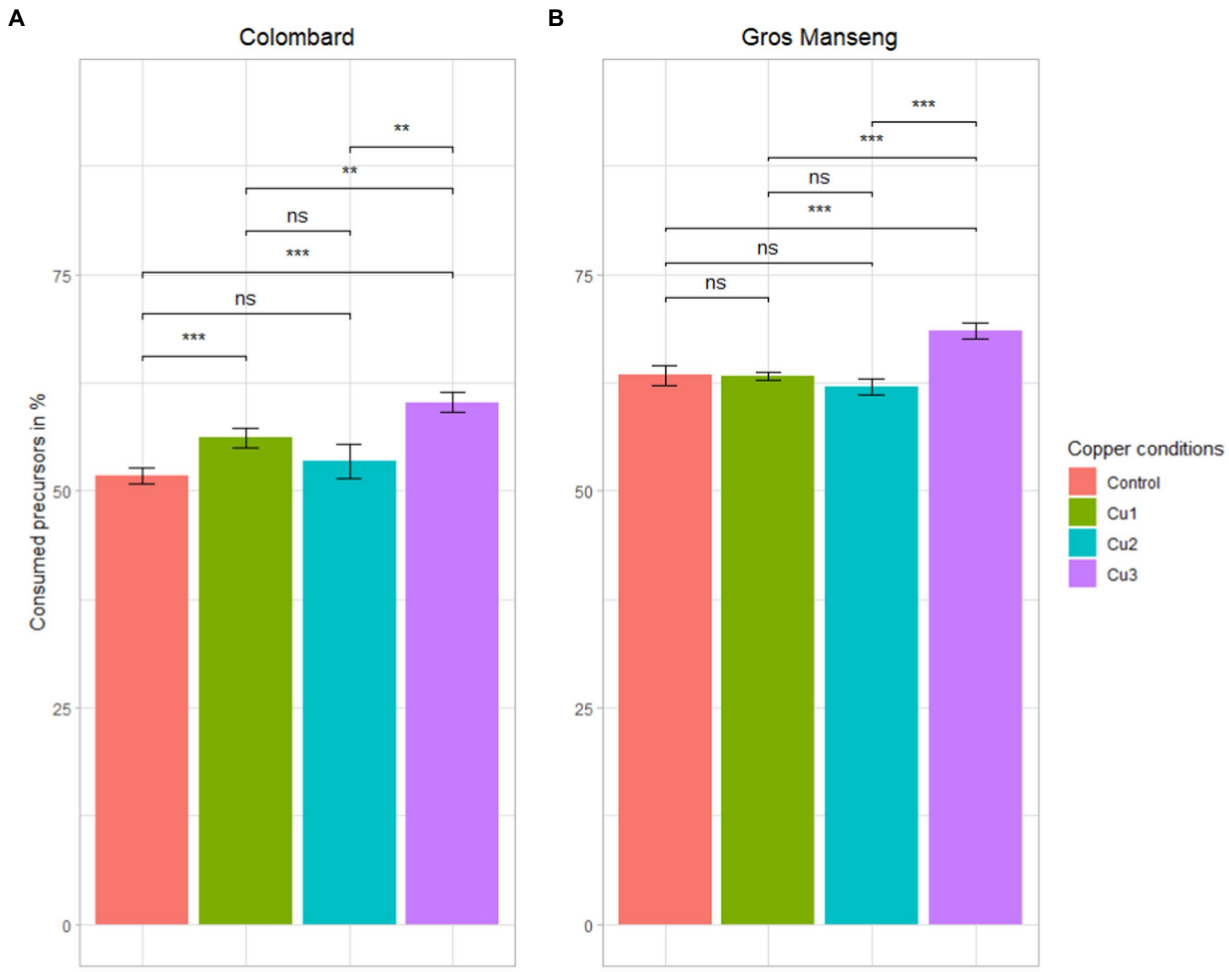
Total precursors consumption in %. **(A)** Colombard **(B)** Gros Manseng. Each plot represents the average of the biological triplicate and the error bars represent the standard errors. value of p were calculated with t-test (95%); * low significant (*p* < 0.1), ** significant (*p* < 0.05), *** highly significant (*p* < 0.01). Copper conditions in mg/L: Colombard control (0.2), Cu1 (0.6), Cu2 (1), Cu3 (3.6); Gros Manseng control (0.4), Cu1 (0.8), Cu2 (1.2), Cu3 (3.9).

Thiol precursors were analyzed at the end of fermentation to assess yeast consumption of these compounds under the different copper levels (Figure 4). The consumption of precursors across all the conditions was in average 55 and 65% for Colombard and Gros Manseng, respectively. These values are lower than the majority of those observed in red grape must of Pinot noir with precursors consumption (G3SH + Cys3SH + CysGly3SH) ranging from 4 to 99.5% with an average of 76% for the 11 AWRI yeast strains studied (Cordente et al., 2022) with an initial content of 1,176, 129.7 and 24.9 μg/l for G3SH, Cys3SH and CysGly3SH, respectively. In Merlot juices with an initial precursor content between 2028 and 9,855 μg/l, consumption ranged from 13 to 47% if considering only Cys3SH and G3SH (Concejero et al., 2016). If we take into account each precursor independently, this latter work suggested that Cys3SH was more assimilated by yeast (over 84%) than G3SH (up to 45%) (Concejero et al., 2016), this last observation corroborating our findings on G3SH assimilation in Colombard and Gros Manseng fermentations where G3SH represented more than 90% of precursors. A similar assimilation of G3SH-d_2,_ around 68%, was measured by Bonnaffoux et al., 2018 using 1,429 μg/l labelled tracers in Sauvignon blanc must with yeast strain Esperide, suggesting that G3SH was possibly never totally assimilated by yeast during alcoholic fermentation in contrast to Cys3SH. All these claims seem to support a mechanism of G3SH uptake that might not be correlated to yeast strain and initial precursors levels in grapes.

For both varieties, the highest precursors consumption (60% for Colombard and 70% for Gros Manseng) was achieved in the ‘Cu3’ condition, which is the highest copper content for both varieties (*p*-values = 0.01 and 0.00012 respectively). This is the first time that an abiotic factor such as copper stress has been evidenced in precursor uptake regulation.

High copper content during fermentation is toxic to yeast that requires several copper resistance mechanisms to maintain homeostasis. One of them starts with Cup2p (also known as Ace1p), a copper-binding transcription factor, that leads to the activation of the *CUP1* gene present in two copies in the *Saccharomyces cerevisiae* genome (Thiele, 1988). This gene codes for a metalothionein, a specific protein that binds copper thanks to numerous cysteine residues (Premakumar et al., 1975). The overproduction of Cup1 proteins containing precisely 12 cysteines in a relatively conserved motif across high eukaryotic cells (Butt et al., 1984) requires an increase in sulfur assimilation and turnover that was later identified in yeast after copper exposure (Yasokawa et al., 2008).

The increased requirement for sulfur might also involve an overproduction of sulfur transporters such as the glutathione transporter Opt1p or the amino acid permease Gap1p. These two transporters have in fact been identified as the main entry mechanism for thiol precursors into yeast (Cordente et al., 2015). It is then possible to attribute the higher consumption of thiol precursors in presence of high copper concentrations to the overproduction of these two transporters.

**Figure 5:**
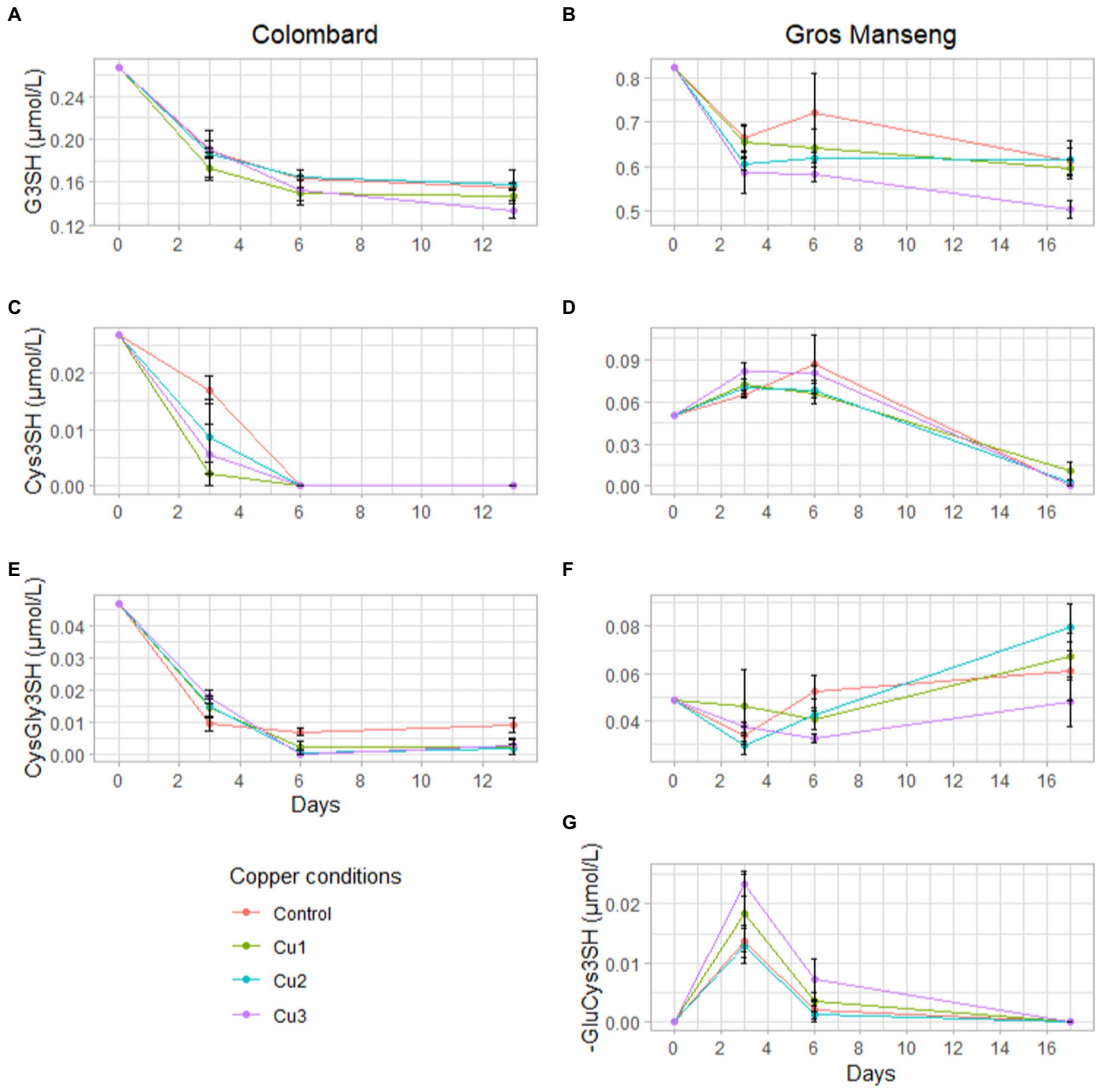
Evolutions of precursor concentration in μmol/L during Colombard (left-side) and Gros Manseng (right-side) fermentations. G3SH in Colombard **(A)** and in Gros Manseng **(B)**; Cys3SH in Colombard **(C)** and in Gros Manseng **(D)**; CysGly3SH in Colombard **(E)** and in Gros Manseng **(F)**; **(G)** γ-GluCys3SH in Gros Manseng. Each point represented the average of biological triplicates and error-bars represented the standard errors. Copper conditions in mg/L: Colombard control (0.2), Cu1 (0.6), Cu2 (1), Cu3 (3.6); Gros Manseng control (0.4), Cu1 (0.8), Cu2 (1.2), Cu3 (3.9).

In addition to global precursors consumption, we also assessed the evolution of precursors content during fermentation with sampling at 3 and 6 days after inoculation (Figure 5).

In Colombard, precursors were consumed throughout fermentation with Cys3SH (Figure 5C) and CysGly3SH (Figure 5E) being completely consumed within 6 days for all conditions except for control fermentations that stabilized at 0.01 μmol/l. Surprisingly, the consumption of G3SH (Figure 5A) was not concomitant with any CyS3SH accumulation as observed in a previous work (Bonnaffoux et al., 2017); this can however be explained by larger sampling intervals under our conditions and by the utilization of a different yeast strain (here strain X5 compared to VIN13 and Esperide). The absence of γ-GluCys3SH might imply that the strain used for these fermentations either do not degrade G3SH *via* this dipeptide but directly into cysteine or CysGly conjugates. All the reported works (Cordente et al., 2015; Bonnaffoux et al., 2018) assumed that precursors are always excreted in the medium by yeast. If we consider that yeast does not excrete all the compounds, it could be possible that γ-GluCys3SH was just not excreted into the fermentation medium, considerably blurring the metabolic mechanisms involved. Both of these hypotheses are consistent with the idea that precursor consumption and degradation are strain-dependent processes and thus cannot easily be generalized.

In Gros Manseng, fermentation showed results more consistent with the literature with an accumulation of Cys3SH (Figure 5D) and γ-GluCys3SH (Figure 5G) correlated with a consumption of G3SH (Figure 5B). The presence of this dipeptide during fermentation confirms the capacity of strain X5 to use this degradation pathway as seen by Bonnaffoux et al. with VIN13 and Esperide strains. Surprisingly, CysGly3SH (Figure 5E) was produced throughout fermentations, leading one to believe that either X5 was capable of producing this dipeptide from the degradation of G3SH, which to our knowledge has never been reported, or that Gros Manseng could harbor unidentified precursors that could be degraded into this dipeptide. Indeed, we can assume that an aldehyde form or sulfite adduct of CysGly3SH might be naturally present in grape juice and could constitute a yet unsuspected precursor pool similar to G3Shal (Thibon et al., 2016; Muhl et al., 2022).

G3SH was the only precursor whose consumption was significantly increased at the highest copper concentration as already seen for total precursors consumption.

The identification of both dipeptides in Gros Manseng fermentation reopened the question about the absence of γ-GluCys3SH in Colombard fermentation and the presence of CysGly3SH. Indeed, we observed a production of γ-GluCys3SH in Gros Manseng (initial G3SH level = 335 μg/l) but not in Colombard (initial G3SH level = 109 μg/l); similarly γ-GluCys3SH was identified in spiked Sauvignon musts (initial level of G3SH-d*_i_* = 579 μg/l) (Bonnaffoux et al., 2018).

As CysGly3SH, γ-GluCys3SH could be the result of the degradation of unknown precursors that may be variety dependent, thus explaining the presence of this dipeptide only in Gros Manseng. Alternatively, considering that γ-GluCys3SH was observed at ‘high’ concentrations of G3SH, the presence of this dipeptide might be linked to the initial level of G3SH in juices. This could correspond to a metabolic route where γ-GluCys3SH would be the result of the overflow of another degradation route (probably CysGly3SH) activated only in presence of high G3SH concentrations.

### 3.4. Varietal thiol revelation

Varietal thiol aromas are extremely reactive compounds that can exist in wine under numerous forms:

- Their reduced form (-SH) in equilibrium with their oxidized (RS-SR’) (Bencomo-Rodriguez et al., 2012) or (R-Sn-R) counterparts (Dekker et al., 2020)
- Complexed with metal such as copper (Franco-Luesma & Ferreira, 2014)
- Oxathianes from the reaction with acetaldehyde (Chen et al., 2018; Wang et al., 2020)
- Thioether bound to polyphenol (Nikolantonaki et al., 2010)

Among these forms, only reduced thiols and oxathianes are odorous compounds participating in the wine organoleptic profile. Oxathianes are found in bottled ageing wine, and correlated with the increase of acetaldehyde content due to oxidation or for some strains, to copper-induced stress during fermentation (Capece et al., 2017; Zimdars et al., 2019). The oxidized and complexed forms can be considered as thiol sinks because they can form back RSH forms during ageing and therefore positively contribute to the wine aroma profile. To the best of our knowledge, there is no method to quantify 3SH and 3SHA copper complexes/products in wines. Consequently, we decided to monitor only reduced and oxidized forms of 3SH and 3SHA according to Roland et al. (2016) approach to obtain a picture of the mechanisms involved under copper stress more complete than through monitoring the reduced forms only. Indeed, the quantification of oxidized forms considered all the combinations of 3SH or 3SHA with other sulfur compounds in the wine matrix, allowing more accurate material balances.

#### 3.4.1. Reduced thiols

**Figure 6:**
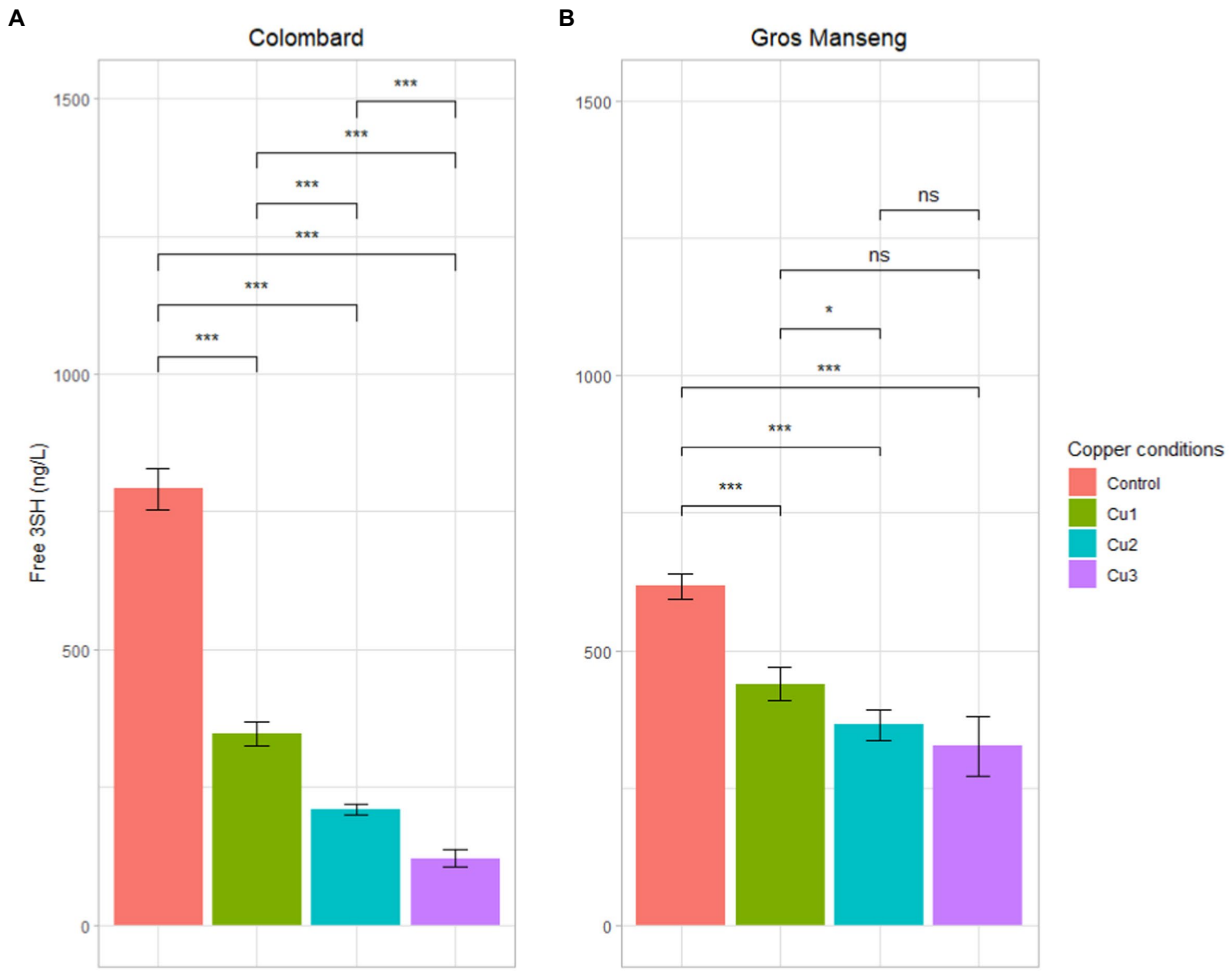
Free 3SH (reduced form) contents in ng/L in all the finished fermentation. **(A)** Colombard **(B)** Gros Manseng. Each plot represents the average of 9 analytical values, errors bars represent the standard error. value of p were calculated with t-test (95%); * low significant (p < 0.1), ** significant (p < 0.05), *** highly significant (p < 0.01). Copper conditions in mg/L: Colombard control (0.2), Cu1 (0.6), Cu2 (1), Cu3 (3.6); Gros Manseng control (0.4), Cu1 (0.8), Cu2 (1.2), Cu3 (3.9). One biological replicate of Gros Manseng Cu3 condition was divergent and therefore excluded of the analysis.

Free 3SH and 3SHA (reduced form) were analyzed in triplicate (*n* = 3) in all biological fermentation replicates s (n = 3) allowing the recovery of a set of 9 data for statistical purpose (Figure 6). 3SHA was detected but remained below the quantification threshold in all samples.

Colombard control conditions produced in average 791 ± 38 ng/l of 3SH against 347 ± 22, 209 ± 10 and 121 ± 15 ng/l for Cu1, Cu2 and Cu3 conditions, respectively. This decrease of 3SH content in wine was significant (value of *p* <1%) and clearly correlated with copper concentrations described in Table 2.

In Gros Manseng fermentation, 3SH production was 617 ± 23, 439 ± 31, 365 ± 28 and 325 ± 56 ng/l for control, Cu1, Cu2, Cu3, respectively. The same trend as the one in Colombard could be observed but the difference between the conditions was less pronounced, only the control was significantly higher than the other conditions (value of *p* <1%). Our observations were consistent with literature: indeed, high copper concentration before fermentation correlated with 3SH content diminution (Darriet et al., 2001). In the present work, 3SH remained twice higher than its perception threshold of 60 ng/l (Tominaga et al., 1998a) for the lowest concentrations in wine. Interestingly, up to 3.6 and 3.8 mg/l of copper in Colombard and Gros Manseng juices respectively, the thiol content of the experimental wines was technically sufficient to contribute perceptibly to the organoleptic profile.

In addition to the obvious chemical oxidation of thiols in the presence of copper, we can hypothesize a biological effect of copper on the cleavage of thiol precursors. As explained above, copper resistance is based upon copper sensing with the transcription factor Cup2p and copper sequestration by protein Cup1p. Another mechanism also used by *Saccharomyces cerevisiae* is Mac1p regulation (Jungmann et al., 1993). Similarly, to Cup2p, this protein is a transcription factor susceptible to regulate copper. In contrast to Cup2 that is activated at high copper contents, Mac1p is inhibited in copper-replete cells (Keller et al., 2005). The targets of Mac1p regulation are many; among those, gene IRC7 is of particular interest in the case of thiol production. Indeed, this gene codes for a β-lyase enzyme responsible for the last step of precursor degradation s (Thibon et al., 2008). Following this regulation by Mac1p, it has been shown that IRC7 transcription level decreases at high copper concentrations (Gross et al., 2000). This decrease results in a lower enzyme content and therefore in a potentially lower thiol release during fermentation performed at high copper concentrations.

#### 3.4.2. Total thiols

**Figure 7:**
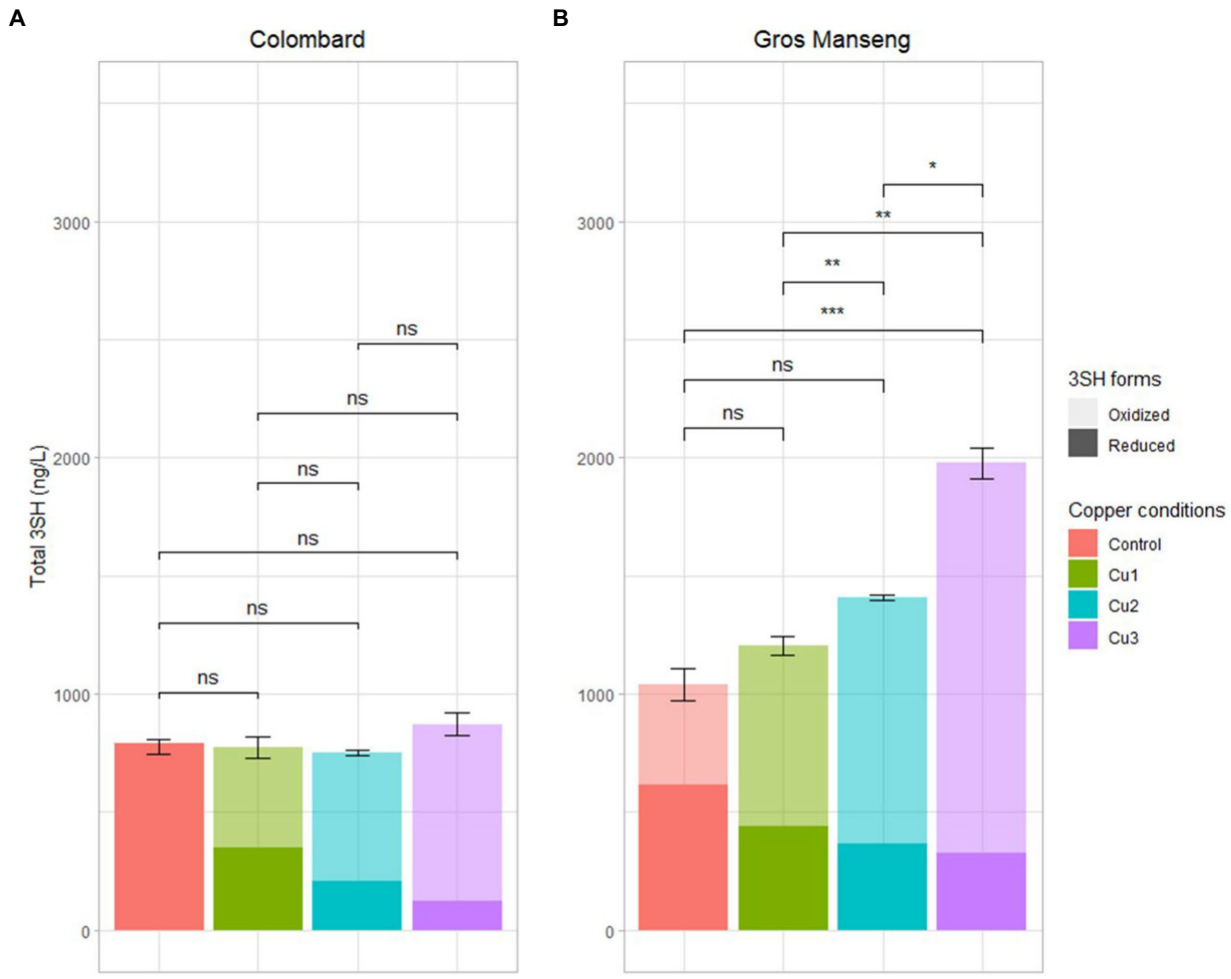
Total (reduced + oxidized) 3SH content in ng/L analyzed with TCEP method in all the finished fermentations **(A)** Colombard and **(B)** Gros Manseng. Each plot represents the average of 3 values (biological replicates), errors bars represent the standard error. value of p were calculated with t-test (95%); * low significant (p < 0.1), ** significant (p < 0.05), *** highly significant (p < 0.01). Copper conditions in mg/L: Colombard control (0.2), Cu1 (0.6), Cu2 (1), Cu3 (3.6); Gros Manseng control (0.4), Cu1 (0.8), Cu2 (1.2), Cu3 (3.9). Control and Cu3 conditions for Gros Manseng were only duplicated.

As explained at the beginning of this section, analysis of the reduced form of thiol aromas is of relevance for industrial purposes but it does not take into account all derived forms of 3SH. To explore all these different hypotheses, the analysis of total thiols (Roland et al., 2016) was carried out to access the total production of thiols during fermentation (Figure 7).

Colombard presented similar levels of total 3SH (Figure 7A) with an average of 794 ± 21 ng/l across all copper conditions tested in this work. Considering the difference observed for precursors consumption, conversion yield for total aroma (reduced + oxidized) and all precursors were in average, respectively, 3.282 ± 0.001, 3.013 ± 0.001, 3.123 ± 0.001, 3.184 ± 0.001% for ‘Control’, ‘Cu1’, ‘Cu2’, ‘Cu3’ modalities for Colombard. These figures are in the upper range of those encountered in natural conditions, i.e., from 0.08 to 4.4% (Roland et al., 2011b; Bonnaffoux et al., 2018; Cordente et al., 2022), even if to date comparisons with other research works remain difficult as calculation methods for conversion yield are not always identical. Based on the results presented here, copper could not be correlated with thiol conversion yield during fermentation from a yeast metabolism point of view. Nevertheless, copper influences level of thiols on both the reduced and the oxidized forms and modulates wine aroma profile from a winemaking point of view.

Reduced forms of 3SH are fully offset by an oxidized fraction inversely proportional to copper concentration, leading to a similar level of 3SH being produced. For Colombard, the reduced form of 3SH is therefore solely correlated to the chemical effect of copper as biological aspects (conversion and total production) remained constant across all conditions.

Fermentation of Gros Manseng (7B) showed even more surprising results with an increase in total 3SH content along with copper concentration increase. This difference was most pronounced for the ‘Cu3’ condition (1977 ± 63 ng/l), which is significantly higher than the other three conditions: the control (1,041 ± 67 ng/l, value of *p* <1%), ‘Cu1’ (1,204 ± 41 ng/l, value of *p* <5%), ‘Cu2’ (1,406 ± 11 ng/l, value of *p* <10%). For this variety, the conversion yield increased with copper content increase with 0.657 ± 0.001, 0.774 ± 0.001, 0.922 ± 0.001, 1.149 ± 0.001% for the ‘Control’, ‘Cu1’, ‘Cu2’, ‘Cu3’ conditions, respectively. These values are relatively low compared to the literature already cited for Colombard. The increased thiol production could be quite simply explained by the increase in precursors consumption, linking copper content to a possible biological modulation of precursors revelation by yeast during fermentation. Further, considering the pattern of precursors evolution during the fermentation of Gros Manseng must, it cannot be excluded that this must could contain unknown precursors whose conversion could be enhanced by copper. However, increased copper concentrations in grape must have been shown to decrease Adh1p and Adh2p activity in *Saccharomyces cerevisiae* during alcoholic fermentation (Zimdars et al., 2019). This reduced activity rules out the possible existence of unknown aldehyde precursors as a key step for this kind of precursors is the reduction of the aldehyde function to an alcohol.

#### 3.4.3. Oxidized thiols

**Figure 8:**
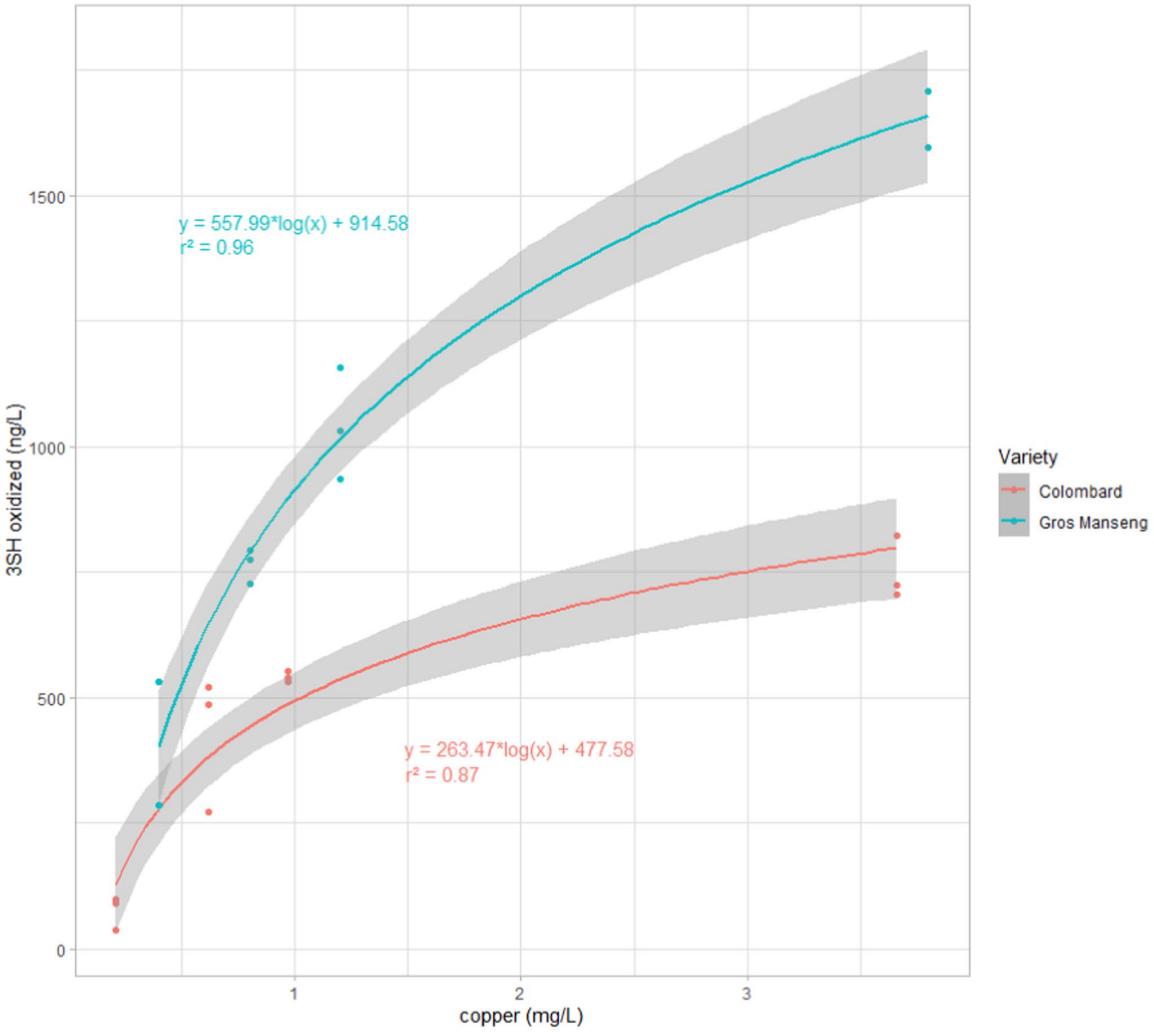
3SH oxidized forms for each copper modalities in both grapes varieties (Red) Colombard, (Blue) Gros Manseng. For each variety a logarithmic fitting was performed. The grey area around each regression represents the standard error of the fitting model.

When considering only oxidized thiols, a clear correlation with copper content appeared, which was then plotted (Figure 8) and a regression model was tentatively applied.

The two different logarithmic regression models applied for each varieties (Figure 8) revealed high correlation coefficients of 0.87 and 0.96 for Colombard and Gros Manseng, respectively.

As to Colombard, the ratios between the 3SH oxidized fraction and 3SH total production are 10 ± 4%, 54 ± 13%, 72 ± 3%, and 86 ± 5% for control, Cu1, Cu2, Cu3 modalities, respectively, while in Gros Manseng, the results in the same order are 39 ± 13%, 64 ± 6%, 74 ± 7%, and 84 ± 8%. With the exception of the controls, it seems that this ratio is not dependent on the quantity of thiols but rather on the quantity of copper. Surprisingly, this ratio does not seem to be correlated with the grape variety.

Thus, as the production of thiols increases with must copper content, at least for Gros Manseng, it would seem that it is possible to compensate for the presence of copper in the environment and to obtain aromatically powerful wines (thiol-wise) by producing more thiols through the management of yeast strain and fermentation parameters.

## 4. Conclusion

In this work, we have evidenced the multiple effects of copper on varietal thiol aromas during wine fermentation. For the first time, copper was shown to have an important oxidation effect on varietal thiols during fermentation, which had previously been observed in wine ageing. Furthermore, new insights on precursors consumption by yeast during fermentation bring into light a biological effect of copper in cells by increasing precursors uptake. Moreover, in Gros Manseng wines, the analysis of total 3SH revealed a completely unsuspected increase in 3SH production by yeast under high copper concentration, strongly suggesting the presence of control pathways or even the existence of still unknown precursors. The absence of such effect in Colombard wines implies a variety-dependent copper effect that has never been shown before. In terms of methodology, this work revealed the importance of jointly analyzing reduced and total 3SH to better understand studied parameters as well as to avoid any premature conclusion.

Overall, we have shown both a chemical and a biological effect of copper on thiols precursors consumption and interconversion as well as on varietal thiol production and RSH/RSSR’ ratio during alcoholic fermentation.

## Data availability statement

The raw data supporting the conclusions of this article will be made available by the authors, without undue reservation.

## Author contributions

GD conducted the experiment, performed the interpretation of data and drafted the manuscript. TD participated in the fermentation experiment and LS participated in the analyses of samples. GD and TD designed the experiment. AR and JRM conceived the overall study. AR and JRM revised the manuscript. All authors read and approved the final version of the manuscript.

## Funding

We thank the syndicat des Côtes de Gascogne and the Occitanie region for the funding of this thesis.

## Conflict of interest

The authors declare that the research was conducted in the absence of any commercial or financial relationships that could be construed as a potential conflict of interest.

## Publisher’s note

All claims expressed in this article are solely those of the authors and do not necessarily represent those of their affiliated organizations, or those of the publisher, the editors and the reviewers. Any product that may be evaluated in this article, or claim that may be made by its manufacturer, is not guaranteed or endorsed by the publisher.
