# Supplementary material for: Unravelling copper effect on the production of varietal thiols during Colombard and Gros Manseng grape juices fermentation by Saccharomyces cerevisiae
Source: Front Microbiol. 2023 Apr 25;14:1101110. doi: 10.3389/fmicb.2023.1101110 (PMC10167020; doi:10.3389/fmicb.2023.1101110)
Supplement: Supplementary file 1 [file Table_1.DOCX]

Table 1: Basic oenological parameters and 3SH precursors of Colombard and Gros Manseng musts

| **Variety** | **Colombard** | **Gros Manseng** |
| --- | --- | --- |
| **Fructose (g/L)** | 103 ± 10 | 121 ± 12 |
| **Glucose (g/L)** | 105 ± 10 | 112 ± 11 |
| **Ammonium (mgN/L)** | 69 ± 7 | 109 ± 11 |
| **Amino acids (mgN/L)** | 114 ± 11 | 164 ± 16 |
| **Copper (mg/L)** | 0.20 ± 0.02 | 0,40 ± 0.04 |
| **G3SH (µg/L)** | 109 ± 1 | 336 ± 3 |
| **γ-GluCys3SH (µg/L)** | n.d. | n.d. |
| **CysGly3SH (µg/L)** | 13.1 ± 0.1 | 13.6 ± 0.1 |
| **Cys3SH (µg/L)** | 5.9 ± 0.3 | 11.2 ± 0.6 |

*Measure error was standardized at 10% for all analyses except for the precursors for which error represents the relative standard error of the method.*
