# Supplementary material for: Unravelling copper effect on the production of varietal thiols during Colombard and Gros Manseng grape juices fermentation by Saccharomyces cerevisiae
Source: Front Microbiol. 2023 Apr 25;14:1101110. doi: 10.3389/fmicb.2023.1101110 (PMC10167020; doi:10.3389/fmicb.2023.1101110)
Supplement: Supplementary file 2 [file Table_2.DOCX]

Table 2: Concentration of the main fermentation metabolites at the end of fermentation

| **Variety** | **Modality** | **Ethanol (g/L)** | **Fructose (g/L)** | **Glucose (g/L)** | **Glycerol (g/L)** | **Acetate (g/L)** | **Succinate (g/L)** | **Copper (mg/L)** | **Average copper consumption (%)*** |
| --- | --- | --- | --- | --- | --- | --- | --- | --- | --- |
| **Colombard** | **Control** | 95.8 ± 0.4 | 3.1 ± 0.1 | n.d. | 5.1 ± 0.5 | 0.26 ± 0.04 | 2.5 ± 0.3 | 0.20 ± 0.03 | 51 |
|  | **Cu1** | 95.9 ± 1.2 | 2.5 ± 0.1 | n.d. | 5.8 ± 0.1 | 0.28 ± 0.01 | 2.7 ± 0.1 | 0.28 ± 0.01 | 65 |
|  | **Cu2** | 95.3 ± 1.2 | 2.5 ± 0.2 | n.d. | 5.7 ± 0.2 | 0.27 ± 0.01 | 2.7 ± 0.1 | 0.41 ± 0.02 | 65 |
|  | **Cu3** | 91.6 ± 3.3 | 2.7 ± 0.3 | n.d. | 5.5 ± 0.3 | 0.27 ± 0.02 | 2.6 ± 0.1 | 1.52 ± 0.13 | 61 |
| **Gros Manseng** | **Control** | 111.0 ± 0.7 | 6.2 ± 0.4 | 0.5 ± 0.1 | 8.3 ± 0.1 | 0.76 ± 0.01 | 7.5 ± 0.2 | 0.10 ± 0.02 | 52 |
|  | **Cu1** | 112.9 ± 0.4 | 5.6 ± 0.1 | 0.4 ± 0.2 | 8.2 ± 0.1 | 0.75 ± 0.02 | 7.5 ± 0.1 | 0.18 ± 0.02 | 70 |
|  | **Cu2** | 112.5 ± 0.3 | 5.9 ± 0.4 | 0.4 ± 0.1 | 8.2 ± 0.1 | 0.75 ± 0.01 | 7.5 ± 0.1 | 0.23 ± 0.01 | 77 |
|  | **Cu3** | 112.6 ± 0.4 | 5.6 ± 0.2 | 0.4 ± 0.1 | 8.3 ± 0.1 | 0.75 ± 0.01 | 7.5 ± 0.1 | 1.02 ± 0.05 | 72 |

*For each value, are presented the average and the standard deviation for the fermentation triplicate. A statistical test using ANOVA produced no significant difference between modalities in each variety. Copper conditions in mg/L: Colombard control (0.2), Cu1 (0.6), Cu2 (1), Cu3 (3.6); Gros Manseng control (0.4), Cu1 (0.8), Cu2 (1.2), Cu3 (3.9).*

**data referred to the consumption of copper at the end of AF.*
